# Age-dependent emergence of neurophysiological and behavioral abnormalities in progranulin-deficient mice

**DOI:** 10.1186/s13195-019-0540-x

**Published:** 2019-10-22

**Authors:** Dávid Nagy, Lauren Herl Martens, Liza Leventhal, Angela Chen, Craig Kelley, Milan Stoiljkovic, Mihály Hajós

**Affiliations:** 1 Translational Neuropharmacology, Section of Comparative Medicine, Yale University School of Medicine, 310 Cedar St., New Haven, CT 06520 USA; 2 FORUM Pharmaceuticals, Inc., Waltham, MA 02451 USA

**Keywords:** Frontotemporal dementia, Progranulin, Electrophysiology, Reward-seeking/processing, Prefrontal cortex

## Abstract

**Background:**

Loss-of-function mutations in the progranulin gene cause frontotemporal dementia, a genetic, heterogeneous neurodegenerative disorder. Progranulin deficiency leads to extensive neuronal loss in the frontal and temporal lobes, altered synaptic connectivity, and behavioral alterations.

**Methods:**

The chronological emergence of neurophysiological and behavioral phenotypes of *Grn* heterozygous and homozygous mice in the dorsomedial thalamic—medial prefrontal cortical pathway were evaluated by in vivo electrophysiology and reward-seeking/processing behavior, tested between ages 3 and 12.5 months.

**Results:**

Electrophysiological recordings identified a clear age-dependent deficit in the thalamocortical circuit. Both heterozygous and homozygous mice exhibited impaired input-output relationships and paired-pulse depression, but evoked response latencies were only prolonged in heterozygotes. Furthermore, we demonstrate firstly an abnormal reward-seeking/processing behavior in the homozygous mice which correlates with previously reported neuroinflammation.

**Conclusion:**

Our findings indicate that murine progranulin deficiency causes age-dependent neurophysiological and behavioral abnormalities thereby indicating their validity in modeling aspects of human frontotemporal dementia.

## Background

Frontotemporal dementia (FTD) is the second most frequent cause of dementia and one of the most common forms in patients under the age of 65 [1]. FTD is clinically defined by progressive changes in behavior and personality, language deficits, cognitive decline, and eventual death [2]. Pathologically, FTD is characterized by focal brain mass loss in the frontal and temporal lobes, hypoperfusion in the affected brain regions, gliosis, neuronal inclusions of various aggregated proteins, and neuronal loss in the affected regions [3, 4]. Approximately 50% of FTD cases have a familial origin with mutations in *MAPT*, *GRN*, and *C9ORF72* causing the majority of these cases [2, 5].

Mutations in the progranulin (*GRN*) gene were causatively linked to FTD in 2006 and account for 5–10% of all FTD cases [6–8]. Disease-causing mutations span the gene and result in one non-functional allele, thereby making the mutations loss of function [8]. The resulting ≥ 50% reduction in the systemic protein levels indicates that progranulin (PGRN) haploinsufficiency is causative for FTD-GRN [9]. Pathology associated with FTD-GRN includes TDP-43 aggregates (TDP type 1) [10, 11]. In MRI imaging studies, FTD-GRN patients have consistent asymmetric frontal, temporal, and parietal lobe volume reductions [12]. FTD-GRN also presents with a significant amount of neuroinflammation [13]. Interestingly, patients carrying two mutant *GRN* alleles present with adolescent-onset neuronal ceroid lipofuscinosis (NCL), a lysosomal storage disorder [14]. Recently, it has been demonstrated that *Grn*^+/−^ mutation carriers with FTD also have lysosomal abnormalities and increased lipofuscin in their retinas, lymphoblasts, fibroblasts, and postmortem brain tissue [15]. The fact that differences in systemic PGRN levels result in different diseases, including FTD, NCL [16], and cancer [17], indicates that PGRN levels require strict regulation.

Progranulin, a secreted growth factor, plays roles in multiple cellular processes including development, cell survival, inflammation, and wound repair [17]. In the CNS, PGRN is expressed by neurons and microglia and functions as a neurotrophic factor and inflammatory mediator [18]. Multiple mouse models of progranulin deficiency have been generated and characterized in order to begin to understand the CNS functions of PGRN, as well as determine their utility for preclinical modeling of FTD [19–21].

As to whether the *Grn*^+/−^ mice model human FTD-GRN has not been extensively investigated. It is clear that *Grn*^−/−^ mice develop age-dependent neuropathology in the thalamus, hippocampus, and cortex that includes gliosis and ubiquitin-positive aggregates [19, 20, 22], whereas the *Grn*^+/−^ mice do not develop any frank neuropathology, even at 2 years of age [19]. Each mouse model has been extensively tested in batteries of behavioral assays; however, deficits in social interactions are the one consistent test where reductions are observed across different models [19, 20, 22]. It has been demonstrated that the *Grn*^+/−^ mice have reduced social behavior, as well as social dominance abnormalities, which has been linked to dysfunction in the amygdala and prefrontal cortex [19, 23].

In this study, we investigated whether alterations in the underlying neuronal physiology are associated with regions of neuropathology in the PGRN-deficient mice. We focused on the thalamocortical circuitry, which is known to be affected in FTD and has been reported to be associated with complement-mediated synaptic loss in *Grn*^−/−^ mice [24]. The dorsomedial (DM) thalamus receives input and sends major outputs to the amygdala, the region of the limbic system most often associated with emotional and social behavior, as well as to the prefrontal cortex, a region associated with executive function and behavioral inhibition [25]. Lesion studies in rats and monkeys have concluded that damage to the DM thalamus is linked to problems with behavioral flexibility and deficits in developing new behavioral strategies to obtain rewards [25]. In fact, FTD patients are known to have deficits in reward-seeking behaviors such as overeating, hypersexuality, and alcohol abuse [26, 27], and these deficits were linked to impairment of the thalamocortical feedback loop [28]. Therefore, based on the profound thalamic neuropathology observed in proganulin-deficientmice and the potential translatability of this region to the FTD phenotype, the current set of studies will focus on neurophysiological and behavioral paradigms that evaluate the integrity of thalamocortical functioning.

## Methods

### Animals

To investigate the chronological emergence of the neurophysiological or behavioral abnormalities in PGRN-deficient animals, we tested three different age groups [3-, 6.5- (neurophysiology only), 9- (behavior only), 12.5-month-old] male 013174 C57BL/6-Grn/J age-matched wild type (WT), *Grn*^+/−^ and *Grn*^−/−^ mice (*n* = 85). All procedures were conducted in accordance with an approved Institutional Animal Care and Use Committee protocol by Yale University or FORUM Pharmaceuticals and with the principles contained in the US Guide for the Care and Use of Laboratory Animals (NIH Publication No. 80–23, revised 1996).

### In vivo electrophysiology

Mice were anesthetized using urethane (1.5 g/kg i.p. injection) and placed on a heating pad (Physitemp Instruments, Inc., Clifton, NJ) set to maintain body temperature at 37 ± 0.5 °C. After achieving the proper level of anesthesia, the heads of the animals were fixed in a stereotaxic frame (Tujunga, CA), and craniotomies were performed above the region of the medial prefrontal cortex (mPFC) and the mediodorsal thalamic nucleus (MD). A stainless steel concentric bipolar electrode (NE-100X, Rhodes Medical Instruments, Woodland Hills, CA) was placed into the mPFC (AP, + 2 mm; ML, − 0.5 mm; DV, − 1.5 mm from Bregma) to record spontaneous local field potentials and evoked field potentials (EFP). An identical electrode was inserted into the ipsilateral MD (AP, − 1.58 mm; ML, 0.44 mm; DV, − 3.2 mm from Bregma) for electrical stimulation. Spontaneous brain activity was monitored for stabilization prior to the start of the recordings. EFPs were amplified (× 1000) and filtered (0.1–100 Hz) using Grass P55 AC differential amplifier (Grass Technologies, West Warwick, RI, USA). The signals were digitized at a rate of 1 kHz through a CED Micro1401-3 interface (Cambridge Electronic Design, Cambridge, UK) and displayed and recorded for online and offline analysis with Spike2 software (Cambridge Electronic Design, Cambridge, UK). Electrically evoked potentials were determined by measuring the difference between the typical positive and negative deflection after stimulation. The electrical stimuli consisted of 0.3-ms-long square pulses, with an inter-stimulus interval of 10 s and were delivered by an Isoflex stimulus-isolator (A.M.P.I. Instruments, Jerusalem, Israel). The basal synaptic properties were tested by generating input-output (IO) curves by recording EFP responses in the mPFC in response to gradually increasing stimulating currents (0–0.3 mA, in 0.02 mA steps) delivered to the ipsilateral MD thalamus. For quantification of genotype-dependent changes shown in IO curves, EFPs of 18 sweeps were generated at each stimulus intensity. The amplitudes of these EFPs were determined and normalized to the maximal response of the WTs at the given age group. Furthermore, to investigate the conductance of the thalamocortical pathway, the latencies of the first positive deflection (~P10) of EFPs were determined using the average of 30 sweeps acquired by 0.2-mA stimuli. The paired-pulse protocol consisted of 2 consecutive current pulses with a range of inter-stimulus intervals (ISI) from 25 to 200 ms. Values of the paired-pulse ratio (PPR) were obtained from the amplitude of the second to the first EFPs. After the conclusion of the experiments, the animals were euthanized, and the brains were rapidly removed and frozen for histological verification of electrodes placement.

### Electrophysiology data analysis

In the IO curves and PPRs, differences among the groups were assessed by one-way ANOVA multiple comparisons followed by post hoc LSD test. Changes in the latency between the groups were assessed using two-tailed*t* tests. Data are expressed as means ± SEM. All data was analyzed using Origin 9.1 (OriginLab Corporation, Northampton, MA, USA), and differences were considered significant when *p* < 0.05.

### Reward-seeking/processing behavior

Reward-seeking/processing behavior was assessed by evaluating sugar pellet consumption in a satiated state. Specifically, the assay evaluated the consumption when the mice are exposed to a palatable food (i.e., sugar pellets). Testing occurred at the beginning of the light cycle at a time when non-fasted mice should be relatively satiated. Testing took place over 2 days: day 1 is pellet pre-exposure to eliminate novelty neophobia, and day 2 is test day. On day 1, mice were fasted overnight and placed in an empty mouse cage with no bedding and exposed to sugar pellets (F06022 Dustless Precision Pellets, 14 mg sugar, Bioserv, Flemington, NJ) placed in a petri dish. Following pellet exposure, mice were returned to their home cage and placed back on ad lib feeding. On day 2, fasted mice were weighed and then placed in the test cage with a pre-weighed petri dish containing sugar pellets and allowed free feed of the pellets. Pellet consumption was assessed by petri dish and spill weight at 30, 60, and 120 min.

For the 9-month-old mice, 1 week following reward-seeking/processing testing, blood glucose levels were measured following an overnight fast at the beginning of the light cycle, similar to the behavioral testing. Blood glucose levels were measured via a drop of blood using a commercially available kit (Freestyle Lite, Abbott).

### Behavior data analysis

Cumulative pellet consumption (mg) over the test period was analyzed. Statistical significance was determined using a one-way ANOVA, followed by post hoc analysis using Tukey’s test; criterion for significance was set at *p* < 0.05.

## Results

### Electrophysiology

**Fig. 1:**
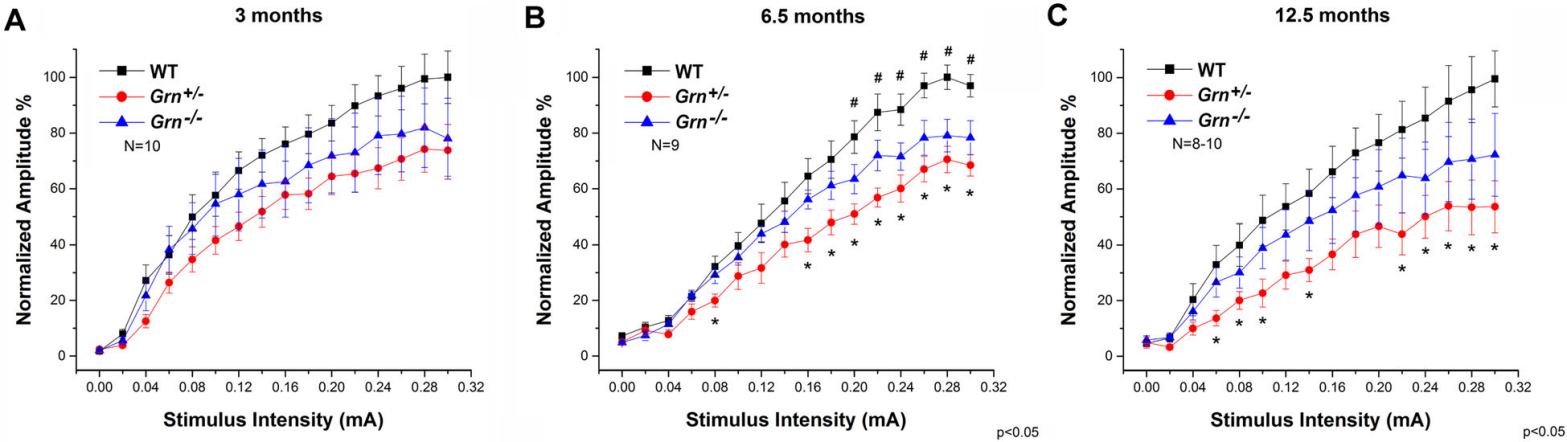
Dorsomedial thalamic neuron deficits in progranulin-deficient mice. Input-output (I/O) curves showing a normalized amplitude of evoked field potentials (EFPs) as a function of stimulation current intensity. **a** I/O relationships at 3 months. **b** I/O relationships at 6.5 months. **c** I/O relationships at 12.5 months. *EFP amplitudes for heterozygotes (*Grn*^+/−^) which are significantly lower than those of WT mice. ^#^EFP amplitudes for homozygotes (*Grn*^−/−^) which are significantly lower than those of WT mice. Data are expressed as the mean ± SEM. Differences in normalized amplitude were considered significant when *p* < 0.05

The input-out (I/O) curves demonstrated a clear current dependent increase in the evoked field potential (EFP) amplitudes up to 0.3 mA, where the responses have reached their maximum in most cases (Fig. 1a–c). Both *Grn*^+/−^ and *Grn*^−/−^ animals exhibited lower amplitudes than WT at each observed age. For homozygous mice, the maximum amplitude was 22% lower compared to WT at 3 months (Fig. 1a); 22% at 6.5 months (Fig. 1b), which was statistically significant; and 28% at 12.5 months (Fig. 1c), which was highly suggestive, but not significant due to higher amplitude variation than the previous ages. The increased variation in amplitudes in the 12.5-month-old mice is most likely due to the age of the animals and was present in all three genotypes. For *Grn*^+/−^, the maximum amplitude was 26% lower than that of WT mice at 3 months (Fig. 1a), while the maximal amplitudes were 32% and 47% lower at 6.5 and 12.5 months, respectively (Fig. 1b, c), both of which reached significance (*p* < 0.003). This data suggests that there is an underlying deficit in the thalamic projection neurons that is both age and gene dose-dependent albeit unexpectedly with the *Grn*^+/−^ mice exhibiting a milder effect than the knockout mice.

**Fig. 2:**
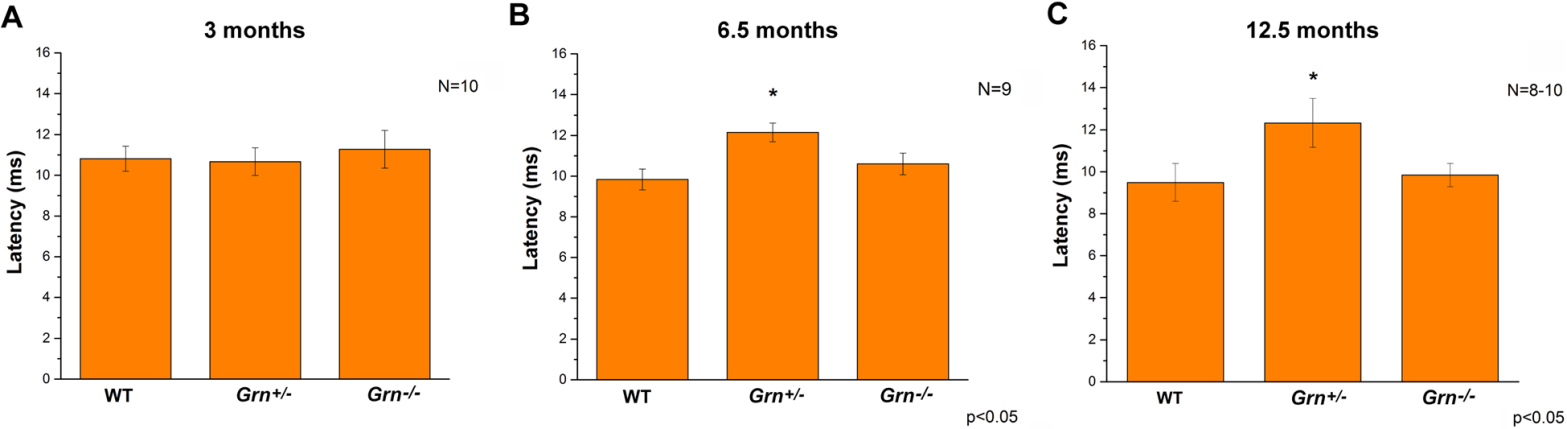
Impaired conductivity of the MD-mPFC pathway in progranulin-deficient mice. The latency of the first positive deflection (P10) in EFPs for WT, heterozygous (*Grn*^+/−^), and homozygous (*Grn*^−/−^) mice. **a** No significant differences in latency among the genotypes at 3 months. **b**, **c** Latency was significantly greater in heterozygous mice at 6.5 and 12.5 months. Data are expressed as the mean ± SEM. Differences in normalized amplitude were considered significant when p < 0.05

**Fig. 3:**
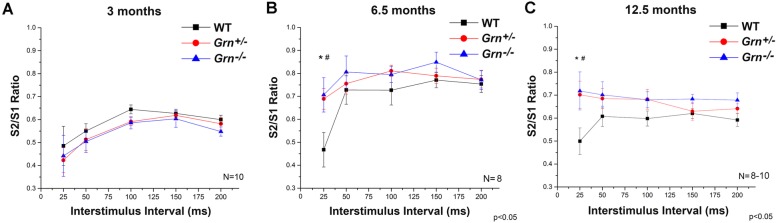
Deficits in feedforward inhibitory signaling to the cortical neurons in progranulin-deficient mice. Paired-pulse inhibition showing the ratio of the second to the first EFPs using 25-, 50-, 100-, 150-, and 200-ms ISIs. **a** No significant differences in PPRs among the genotypes in 3 months. **b**, **c** PPR were significantly lower in both heterozygotes (*Grn*^+/−^) and homozygotes (*Grn*^−/−^) compared to WT using 25-ms ISI; meanwhile, there were no significant differences among the genotypes in the 50–200-ms ISI range. Data are expressed as the mean ± S.E.M. *Significant difference between heterozygotes and WT; ^#^Significant difference between homozygote and WT. Differences in PPRs were considered significant when *p* < 0.05

From measures of conductance, there was no observed age-related changes in either WT or *Grn*^−/−^ (Fig. 2a–c), but latency was significantly higher (*p* < 0.04) in *Grn*^+/−^ mice at 6.5 and 12.5 months of age (Fig. 2b, c). This alteration indicates impaired conductivity of the mediodorsal thalamic nucleus (MD)-medial prefrontal cortical (mPFC) pathway. The MD-driven thalamocortical feedforward inhibition onto the mPFC pyramidal neurons showed paired-pulse depression as indicated by a ratio < 1 in all experimental groups (Fig. 3). There were no significant differences in the paired-pulse ratio (PPR) among the phenotypes at 3 months of age in any tested inter-stimulus interval (ISI) (Fig. 3a). Both *Grn*^+/−^ and *Grn*^−/−^ animals indicated altered PPR with 25-ms ISI at 6.5 and 12.5 months which were significantly lower than those in WT (Fig. 3b, c), whereas there were no significant differences in the 50–200-ms ISI range among the phenotypes in any age groups (Fig. 3). Together, these data indicate age-dependent deficits in local cortical circuitry as well as overall impairment of the thalamocortical network in progranulin-deficient mice.

### Reward-seeking/processing behavior

**Fig. 4:**
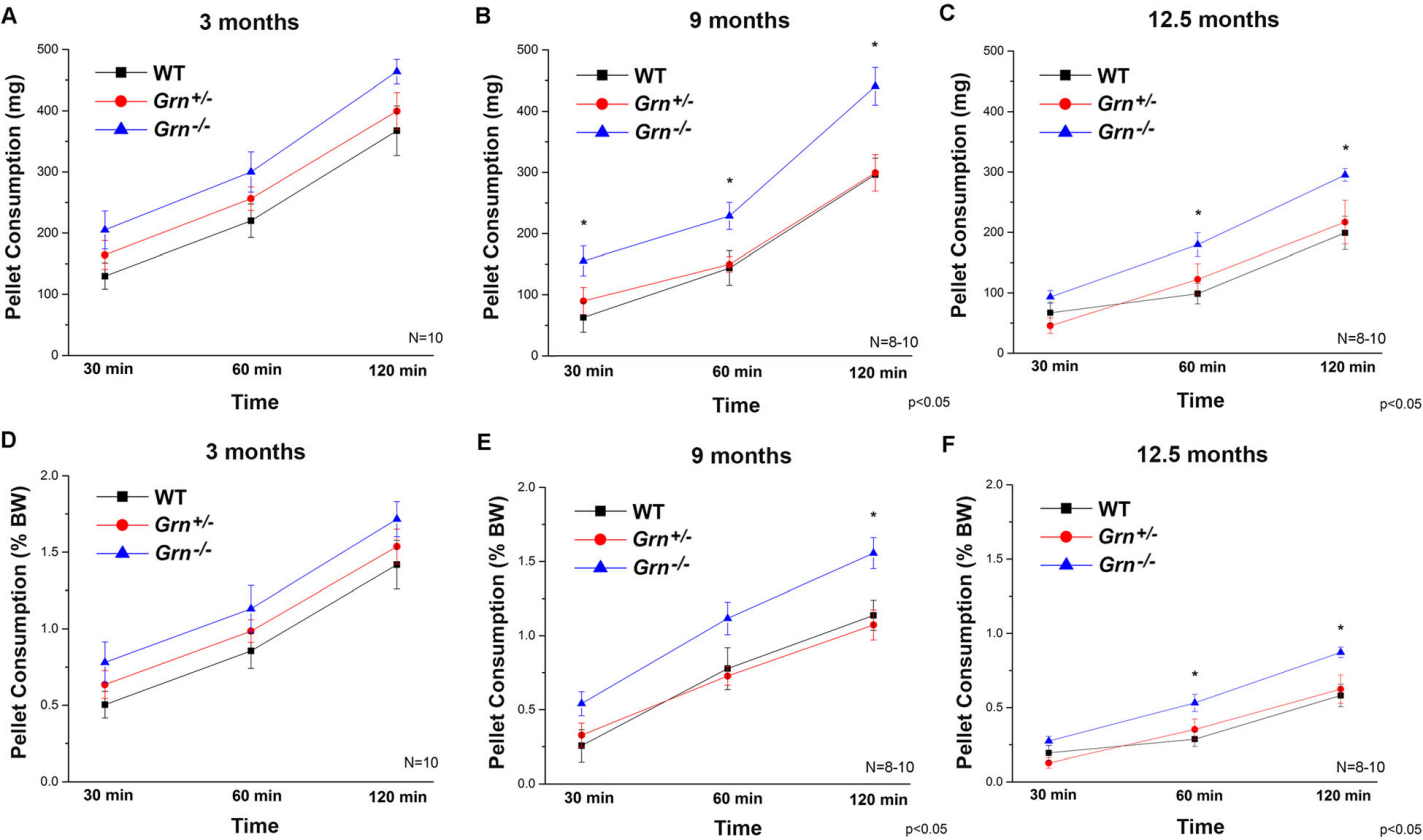
Reward-seeking/processing behavior is only altered in progranulin knockout mice. Cumulative sugar pellet consumption over 120 min [in mg (**a**–**c**) or % body weight (BW) (**d**–**f**)]. **a**, **d** At 3 months. **b**, **e** At 9 months. **c**, **f** At 12.5 months. Data are expressed as mean + SEM. *Significant difference between homozygotes (*Grn*^−/−^) and WT (*p* < 0.05)

Reward-seeking/processing behavior was assessed by evaluating the genotype differences in sugar pellet consumption at 3, 9, and 12.5 months of age. At 9 months (Fig. 4b, e) and 12.5 months (Fig. 4c, f), statistically significant increases (*p* < 0.05) in cumulative pellet consumption were observed in *Grn*^−/−^ compared to WT mice. No significant differences were observed at 3 months, but a numerical increase in the *Grn*^−/−^ is present at this time point (Fig. 4a, d). In contrast, at all time points tested the *Grn*^+/−^ mice did not significantly differ from WT mice.

In order to determine if the observed differences could result from glucose metabolism alterations, blood glucose levels were measured in the 9-month-old mice 1 week following testing. No significant difference in the blood glucose level was observed between WT, *Grn*^+/−^, and *Grn*^−/−^ mice (128.1 ± 9.1, 135 ± 8.4, and 125 ± 5.9 mg/dL, respectively). Similarly, at 3, 9, and 12.5 months of age, no significant difference in the body weight was observed between the groups (data not shown). The present results demonstrate that impairment of the thalamocortical circuitry in the *Grn*^+/−^ mice is necessary, but not sufficient to cause changes in behavior. Our data indicate that pathological changes, including inflammation, gliosis, and accumulation of lysosomal storage material associated with the onset of pathology as observed in the *Grn*^−/−^ mice, are required for behavioral deficits.

## Discussion

To date, it has been challenging to identify neuronal abnormalities in murine models of PGRN haploinsufficiency that relate to FTD, therefore complicating their use as an efficacy model for therapeutic development. The present findings demonstrate a striking abnormality in the thalamocortical neurophysiology and behavior in PGRN-deficient mice. These new findings provide the first support for the development of a translational model of FTD-GRN.

It has been reported that the *Grn*^−/−^ mice show reduced synaptic connectivity and impaired plasticity, which significantly precedes neuropathological changes and may be the underlying mechanisms contributing to FTD pathology in patients [22]. Our results indicate that the neurophysiological changes that arise in the MD-mPFC pathway become increasingly apparent with age and are reflected by both behavioral and electrophysiological alterations. It has been demonstrated that *Grn* gene deficiency in mice leads to an age-dependent enhancement of microglia-mediated synaptic pruning in the thalamus [24]. In line with these findings, our data indicate an age-dependent reduction in cortical responses to thalamocortical activation, displaying abnormalities in the I/O curves in both *Grn*^+/−^ and *Grn*^−/−^ mice. These observations are in line with behavioral deficits, since both *Grn*^*+*/*−*^ and *Grn*^−*/*−^ mice develop social deficits and exhibit impaired fear memory, as seen in patients with behavioral variant FTD [19, 20]. However, *Grn*^*+*/*−*^ but not *Grn*^*−*/*−*^ mice showed abnormal social dominance in the tube test, suggesting that progranulin haploinsufficiency has distinct effects from complete progranulin deficiency [23]. Similarly, in our study, the *Grn*^+/−^ mice perform worse at all ages evaluated, reaching significant deficiency starting at 6 months of age. These findings provide a potential in vivo assay for studying abnormalities in neuronal circuitries in progranulin-haploinsufficient animals with relevance to human FTD. The fact that *Grn*^+/−^ showed more profound abnormalities in electrophysiological signals than *Grn*^−/−^ may be due to compensatory mechanisms that are activated during development. Another hypothesis is related to the function of the granulins: it has been postulated that PGRN and its GRN cleavage products have reciprocal functions, at least in wound healing, inflammation, and neuroprotection [29–31]. However, considering the etiology of FTD, other factors beyond PGRN deficiency must be taken into consideration. FTD patients also exhibit pronounced atrophy of specific brain regions, significant astrocytosis, and myelin loss not only in white-matter tracts, but in the cerebral cortex as well [12]. Moreover, it has been reported that PGRN deficiency in *Grn*^−/−^ mice leads to selective and age-dependent loss of parvalbumin^+^ inhibitory synapses [24]. MD neurons synapse directly onto and activate these parvalbumin^+^ interneurons in the mPFC that are the major mediator of a feedforward inhibition, as optogenetic silencing of these interneurons greatly suppressed, and in some cases totally abolished this feedforward inhibition [32]. In line with this observation, our findings demonstrate a diminished paired-pulse inhibition with short ISI beginning at 6.5 months of age in *Grn*^+/−^ and *Grn*^−/−^ mice compared to their age-matched WT controls. Furthermore, silencing local interneurons in the mPFC increased the duration and latency of excitatory postsynaptic potentials (EPSPs), suggesting that parvalbumin^+^ interneurons control the temporal integration window of principal neurons [33]. These findings are in accord with our recent observations where reduced activity in the mPFC increases the latency of EPSPs; however, this abnormality was present only in *Grn*^+/−^ animals.

Additionally, recent evidence suggests that interneurons in the mPFC can modulate decision-making in reward-related behaviors [34], and optogenetic activation of these interneurons in the mPFC alters appetite-driven behavior [35] and biases animals toward overeating of sweets. This overeating of sweets is similar and directly translatable to that observed in FTD patients [36]. Studies have linked these behaviors to alterations in the thalamocortical circuitry using voxel-based morphometry suggesting that deficits in primary reward-seeking in FTD patients may be due to the impairment of the thalamocortical feedback loop [28]. The behavioral task examined in our preclinical studies was reward-seeking/processing by evaluating the consumption of sugar pellets at the beginning of the light cycle time of day when mice should be sated. All groups of mice consumed the palatable treat as expected in this state, but the *Grn*^−/−^ mice consumed a significantly greater amount than the other groups. This increased intake occurred at both time points (9 and 12 months) where neuropathology exists (> 6 months) and the pattern was present, although not significant, pre-pathology (3 months). The increased sugar pellet intake is not the result of the differences in blood glucose levels or body weight (data not shown). Since the *Grn*^+/−^ mice were identical to the WT, the present data suggests that PGRN deficiency alone is not sufficient to elicit this behavior in mice, unlike the observed neurophysiology, but that frank neuropathology or structural changes are necessary to see this behavioral pattern.

## Conclusions

Our findings demonstrate the translatability of PGRN-deficient mice as a model of neuronal dysfunction due to the haploinsufficiency of PGRN. Future studies are needed to determine if similar dysfunction is evident in FTD-GRN patients and whether this preclinical model can be used as a tool for elucidating disease pathophysiology and the development of therapeutics.

## Data Availability

The datasets used and/or analyzed during the current study are available from the corresponding author on reasonable request.

## Abbreviations

AP: Anterior-posterior
C9ORF72: Intronic expansion of a hexanucleotide GGGGCC repeat in the chromosome 9 open reading frame 72
CNS: Central nervous system
DM: Dorsomedial
DV: Dorsal-ventral
EFP: Evoked field potential
EPSP: Excitatory postsynaptic potential
FTD: Frontotemporal dementia
GRN: Granulin
i.p.: Intraperitoneal
IO: Input-output
ISI: Interstimulus interval
MAPT: Microtubule-associated protein tau
MD: Mediodorsa
ML: Medial-lateral
mPFC: Medial prefrontal cortex
NCL: Neuronal ceroid lipofuscinosis
PGRN: Progranulin
PPR: Paired-pulse ratio
TDP-43: Transactive response (TAR) DNA-binding protein with a molecular weight of 43 kDa
WT: Wild type
